# Tailored management of cavernous malformations in women: considerations and strategies—a review

**DOI:** 10.3389/fneur.2024.1487808

**Published:** 2024-10-24

**Authors:** Delal Bektas, Giuseppe Lanzino, Kelsey M. Smith, Kelly D. Flemming

**Affiliations:** ^1^Department of Neurosurgery, Mayo Clinic, Rochester, MN, United States; ^2^Department of Neurosurgery, University Hospital Zurich, Zurich, Switzerland; ^3^Department of Neurology, Mayo Clinic, Rochester, MN, United States

**Keywords:** cavernous malformation, female, women, seizure, epilepsy, estrogen, progesterone, management

## Abstract

**Purpose of review:**

Cavernous malformations (CM) are vascular lesions in the brain and spinal cord, characterized by clusters of endothelial-lined caverns lacking proper tight junctions. These malformations may be discovered incidentally or present with symptoms such as headaches, focal neurologic deficits, or seizures, with or without hemorrhage. This review focuses on non-surgical management considerations important for women with CM, who face challenges related to pregnancy, exogenous hormone use, anticonvulsive therapy, bone health, and mental health.

**Recent findings:**

Emerging evidence suggests that both estrogen and progesterone may influence CM lesion behavior. Exogenous hormones, including those in oral contraceptives and oral hormone replacement therapy, indicate an elevated risk of symptomatic hemorrhage (SH) and may also influence seizure frequency and severity, particularly in women taking antiseizure medications (ASMs). Data suggest that the risk of CM hemorrhage during pregnancy is similar to the risk when not pregnant, although limitations to these studies will be reviewed.

**Summary:**

This review synthesizes the current literature on the interplay between estrogen and progesterone and CM lesion behavior, highlighting the importance of gender- and sex-specific factors in clinical decision-making. Special attention is given to the implications of exogenous hormone use, seizure management, and the psychological well-being of women with CM, underscoring the need for a multidisciplinary approach tailored to the unique needs of this patient population.

## Highlights

- The clinical presentation, size, and location of CM in female patients are similar to men, but sex-specific hormonal influences may impact outcomes and management strategies.
- The risk of CM-associated hemorrhage or seizure during pregnancy is generally low; however, careful preparation, planning, and monitoring are essential to ensure optimal outcomes for both mother and infant.
- Caution is advised when considering oral exogenous estrogen and progesterone in women with CM, as these may increase the risk of SH and influence seizure activity.
- Women with CM and seizure disorders should be informed about the long-term effects of ASMs, including their impact on bone health, contraceptive interactions, and potential effects on a fetus or breastfed infant.
- Addressing mental health is as important as managing physical health in women with CM, requiring comprehensive care.

## Introduction

Cavernous malformations (CM) are vascular lesions primarily in the brain and spinal cord that affect approximately 0.5% of the population (1, 2). These lesions are composed of clusters of dilated capillaries that lack properly formed tight junctions, making them prone to hemorrhage (3–5). CM can be identified incidentally or manifest with seizures, focal neurological deficits, and headaches with or without radiologic evidence of hemorrhage (6). CMs can be classified as either familial or sporadic (7, 8). The familial cerebral cavernous malformation syndrome (FCCM), is typically characterized by multiple lesions and is associated with mutations in the *CCM1* (*KRIT 1*), *CCM2*, and *CCM3* (*PDCD10*) genes (9–11). Conversely, the sporadic form generally involves a single CM, which is frequently associated with a developmental venous anomaly (DVA) (12–14).

The risk of symptomatic hemorrhage (SH), particularly recurrent events, is a major concern for patients with CM, with morbidity increasing after each hemorrhage. Key risk factors include prior SH and brainstem location (15). The risk of recurrent hemorrhage is highest within the first year and tends to plateau after about 2.5 years, underscoring the need for effective management strategies to prevent hemorrhage and reduce associated morbidity (16, 17). The clinical management of CM primarily aims to prevent SH, which is often addressed through surgical excision when appropriate (18–20). In women, however, the management of concurrent conditions including epilepsy, pregnancy planning and pregnancy, and use of oral estrogen and progesterone may be equally as important to consider in the conservative management of CMs (18).

**Figure 1:**
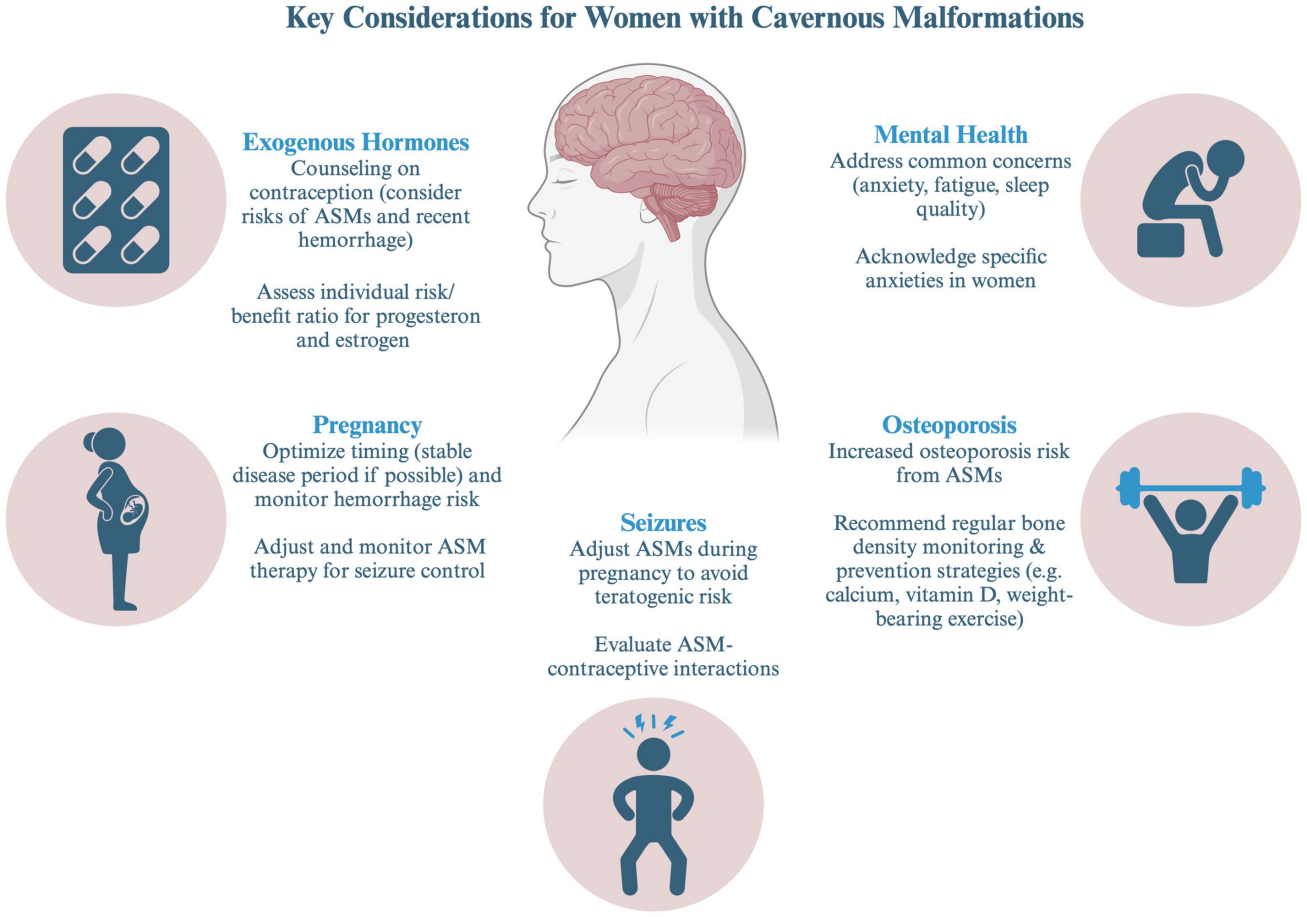
Key consideration for female patients with cavernous malformations (AED, antiepileptic drug). Created in BioRender. Bektas, D. (2024) BioRender.com/u04p136.

This review aims to provide a comprehensive overview of the clinical management considerations in women with CM. A multidisciplinary approach is important to address the specific needs of this population (Figure 1).

## Clinical presentation and hormonal influences in female patients

### Clinical presentation in female patients

Cavernous malformations (CMs) often present in female patients during their reproductive years, with many cases first identified between the second and fifth decades of life, similar to the age group presenting in males (21). Over the past 20 years, numerous cohort descriptions and natural history studies have detailed the initial clinical presentation, and outcomes in patients with CMs (16, 22, 23).

While some studies have suggested female sex as a risk factor for SH, others have not. Aiba et al. followed 110 patients over 4.7 years, finding that females more frequently presented with SH during the second to fifth decades of life, while males more commonly presented with incidental findings (21). Similarly, Robinson observed a significant difference in SH presentation between male and female patients (24). The Scottish Intracranial Vascular Malformation Study (SIVMS) cohort, a population-based study, reported a higher proportion of female patients presenting initially with SH or focal neurologic deficit compared to male patients, although this difference did not reach statistical significance (17). In a study of 68 patients over a 5.2-year period, Moriarty et al. found that female patients experienced a significantly higher hemorrhage rate of 4.2% per patient-year, compared to just 0.9% per patient-year in males, despite having similar initial clinical presentations (25). However, other studies, such as the individual meta-analysis by Horne and colleagues, have identified SH at diagnosis and brainstem location as the primary factors that increase the risk of prospective hemorrhage (15). Whether the differences noted in these cohorts was due to the influence of exogenous or endogenous hormones is not certain.

### Influence of hormonal changes on CM

#### Endogenous hormones

Endogenous hormones, particularly estrogen and progesterone, have been hypothesized to influence CM behavior (26–28). Early studies suggested that women are more likely to present with SH during periods of significant hormonal change, such as pregnancy (21, 24), due to factors such as hyperdynamic circulation, increased turbulent blood flow, and higher estrogen levels leading to endothelial cell degeneration. Also, placental production of growth factors such as VEGF, bFGF, and placental growth factors were hypothesized to contribute to proliferation and angiogenesis, potentially exacerbating CM lesional activity (29) Hormonal changes during pregnancy have also been proposed to cause intralesional CM or DVA thrombosis, leading to venous hypertension and subsequent CM hemorrhage (29, 30). More recent studies, including those by Kalani, Witiw, and Joseph, suggest that the risk of hemorrhage during pregnancy may not differ significantly from the non-pregnant state, but have limitations (31–33). These studies are discussed in more detail under the section “Pregnancy and CM Management.” Although the literature has not specifically addressed the effects of endogenous estrogen on CMs, it has been suggested that estrogen plays a crucial role in maintaining blood–brain barrier integrity and supporting vascular repair. Estrogen may enhance the repair of endothelial cells and help restore the tight junctions within the blood–brain barrier (34, 35), which could potentially mitigate the risk of hemorrhage. However, this protective effect might decrease with aging (36, 37), leading to greater permeability of the blood–brain barrier and an increased susceptibility to hemorrhagic stroke.

Zhang et al. suggest that progesterone may also negatively affect the cerebral cavernous malformation (CCM) complex, which stabilizes the endothelium (26, 28, 38). Recent research has further elucidated the role of progesterone receptors in CCMs, indicating that activation of progesterone receptors can disrupt the blood–brain barrier and exacerbate CM pathology by promoting vascular permeability and inflammation (38). Additionally, the absence or dysregulation of progesterone receptor signaling has been associated with structural changes in the blood–brain barrier, further contributing to the progression of CMs (27).

These data limitations underscore the need for more robust, prospective studies to better understand the relationship between endogenous hormonal fluctuations and CM behavior. Moreover, there remains a need for further investigation into how other hormonal changes, such as those during the menstrual cycle and menopause, may influence CM behavior, as the current literature has predominantly focused on pregnancy and hormone use.

#### Exogenous hormones

Concerns have been raised about the impact of exogenous estrogen and progesterone, such as those found in oral contraceptives and oral hormone replacement therapy (HRT) used for perimenopausal symptoms on CM behavior (39).

A prospective cohort study from the Mayo Clinic found that brainstem location and estrogen use in female patients were associated with an initial presentation of SH, although the study had a small sample size and did not differentiate between hormone delivery methods (40). A larger study by Zuurbier and colleagues analyzed 722 female patients, 137 of whom had taken exogenous estrogen and/or progesterone for contraception or HRT. The study found that 33.6% of patients taking hormones experienced a prospective CM hemorrhage, compared to 15.6% of those not taking hormones. The increased hemorrhage risk was particularly significant for those using oral contraceptives, especially when combined with smoking. In the HRT group, oral hormone replacement, but not other hormone delivery methods were associated with an increased risk of SH. While estrogen-containing contraceptives were associated with an increased risk, the study grouped oral progesterone-only contraceptives together with combined estrogen-progesterone options, rather than evaluating them separately. Additionally, progesterone-only methods delivered via intrauterine devices (IUDs) were not evaluated due to differences in the ascertainment of data between included cohorts. This grouping approach presents a limitation, as it does not allow for a clear assessment of the risks associated with progesterone-only contraceptives.

While the above study raises concerns about exogenous oral estrogen and progesterone in patients with CM, many questions remain. If estrogen increases SH risk by its influence on intralesional thrombosis, then the effect should be dose dependent, dependent on which generation progesterone it is combined with and may be dependent on the mode of delivery (transdermal versus vaginal versus oral). The potential negative effects of progesterone on the CCM complex should still be considered given the work by Zhang and colleagues, but further research is necessary to accurately distinguish the risks of progesterone separated from the estrogen effect (39). A women’s health specialist should be consulted to discuss alternative options such as copper IUDs for pregnancy prevention or consider the lowest risk estrogen/progesterone combination in patients with medical conditions requiring hormones.

For transgender patients undergoing hormone therapy, similar considerations apply. Counseling for transgender patients should address both the need for gender-affirming care and the risks associated with hormone therapy in the context of CM location, symptoms, and treatment options.

In summary, estrogen-containing contraceptives and oral hormone replacement therapies should generally be avoided in women with CM due to the increased risk of thrombosis and CM hemorrhage associated with estrogen-containing therapies. In addition, given the potential effects of progesterone on the CCM complex, these options should be chosen carefully and monitored closely (26, 28). However, given the many uncertainties of the current data and the need for hormonal therapy in certain conditions, a personalized approach should be emphasized in clinical practice. An individual with CM considering hormone therapy should be evaluated to determine their individual risk of SH and the morbidity associated with potential hemorrhage and compare that to the risks, benefits and alternatives to hormone therapy. For example, there are familial CM patients who inherit the gene, but have no lesions or have only lesions that appear on hemosiderin sensitive sequences. The risk in such patients may not be similar to those with a recently symptomatic hemorrhagic event, brainstem lesion, or enlarging CM lesion. Hormone therapy use should be carefully monitored, with individualized care plans to minimize risk while effectively addressing the patient’s symptoms (39).

## Management of seizures in female patients with CM

### Seizure management, AED and hormonal interactions

Seizure management in women with CM involves additional complexities due to the interactions between antiseizure medications (ASMs) and hormonal therapies as well as the influence of pregnancy, which is further discussed in the paragraph about considerations relevant to pregnancy. In addition to the potential negative consequences of estrogen and progesterone on SH risk, there are additional considerations in CM patients with seizures. Many ASMs, particularly enzyme-inducing medications like carbamazepine, phenytoin, and phenobarbital, can reduce the effectiveness of hormonal contraceptives, potentially leading to contraceptive failure. This reduction in contraceptive effectiveness can lead to unintended pregnancies, which is concerning because many ASMs have teratogenic potential, posing risks to the developing fetus (41–43) (Table 1). Conversely, hormonal therapies can decrease serum levels of ASMs like lamotrigine, which may reduce seizure control and necessitate close monitoring and dose adjustments (42, 44, 45). HRT used for perimenopausal symptoms can also influence seizure control. A small randomized controlled study found that oral HRT can significantly worsen seizure control, though this effect was not specifically tested in patients with CMs, necessitating careful consideration when prescribing these therapies (46). Additionally, endogenous hormones, particularly estrogen, are known to be “pro-convulsant,” with some patients experiencing an increase in seizure frequency during periods of high estrogen levels, such as during pregnancy or certain phases of the menstrual cycle (41).

**Table 1:** Risk of congenital malformations and teratogenic effects of antiseizure medications.

Antiseizure medication	Risk of major congenital malformation among registries (57, 59–62)	Most common reported teratogenic effects
Valproic acid	6.7–10.3%	Neural tube defects, hypospadias, cardiac defects, oral clefts
Phenobarbital	5.5–6.5%	Cardiac defects, oral clefts
Phenytoin	2.9–6.4%	Cardiac defects, digit hypoplasia
Carbamazepine	2.6–5.5%	Neural tube defects
Topiramate	3.9–4.3%	Oral clefts
Oxcarbazepine	2.2–3.0%	No evidence of teratogenicity
Lamotrigine	2.0–2.9%	No evidence of teratogenicity
Levetiracetam	0.65–2.8%	No evidence of teratogenicity

These interactions underscore the importance of a personalized approach to managing seizures in women with CM, considering both the effectiveness of seizure control and the potential risks associated with hormonal therapies. Proper counseling on the choice and use of contraception is vital to ensure that neither the efficacy of the contraceptive nor the ASM is compromised, thereby reducing the risk of unplanned pregnancies and ensuring optimal management of epilepsy (42, 47).

### Bone health considerations

Female patients are generally at a higher risk for osteopenia and osteoporosis compared to males (48), and this risk is further elevated in patients with cavernous malformations (CM) who are on enzyme-inducing ASMs. The enzyme-inducing properties of these ASMs can lead to decreased bone density, exacerbating the risk of bone health issues (49–51) (Table 2). Additional risk factors for osteopenia include advancing age, low body weight, and a history of glucocorticoid therapy (52). To manage these risks, regular bone density screening is recommended for female patients with CM. Supplementation with vitamin D and calcium is also advised to help mitigate the risk of osteopenia and osteoporosis (53). In addition to the value of vitamin D supplementation for bone density, recent studies suggest that vitamin D supplementation may also play a role in influencing CM behavior, possibly through its effects on oxidative stress and the gut microbiome (54–56). Therefore, maintaining adequate vitamin D levels is crucial not only for bone health but also for potentially beneficial effects on CM management.

**Table 2:** Effects of antiseizure medications on bone mineral density and osteoporosis prevention recommendations.

Antiseizure medications and bone mineral density	Recommendations (49)
Lower bone mineral density	Osteoporosis Prevention
Benzodiazepine Carbamazepine Gabapentin Oxcarbazepine Phenobarbital Phenytoin Primidone Valproic acid Zonisamide	Monitor calcium and Vitamin D levels 1–2 time per year Calcium 1,200 mg/ Vitamin D 800 IU daily from diet or supplementation Weight bearing exercises 30 min daily Tobacco cessation Moderate or no alcohol intake Moderate or no caffeine Avoid glucocorticoids
Possibly lower bone mineral density Levetiracetam Topiramate	If Osteopenia/Osteoporosis Detected Pharmacologic treatment discussion regarding bisphosphonates Consider switching antiseizure medication if relevant Vitamin D target levels>30 ng/mL.
Do not affect bone mineral density Lamotrigine	

## Pregnancy and CM management

**Figure 2:**
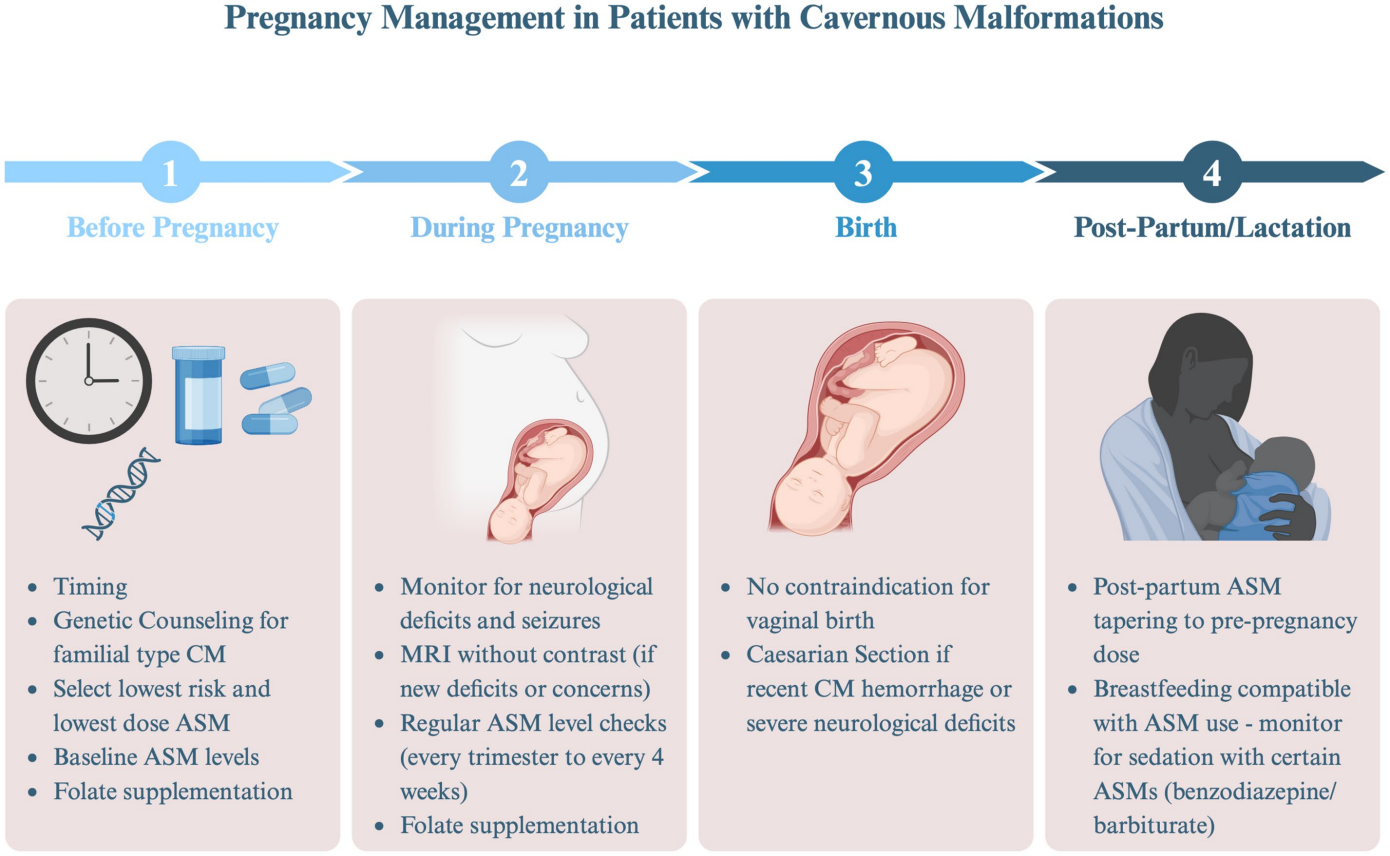
Pregnancy management in patients with cavernous malformations (AED, antiepileptic drug). Created in BioRender. Bektas, D. (2024) BioRender.com/e32g825.

While the absolute risk of SH and seizures during pregnancy are generally low for most patients with CM, there are several factors that require careful consideration (Figure 2).

### Pre-pregnancy planning

Pre-planning and preparation are important for patients with CM who are considering pregnancy. Discussions should include optimal timing of conception relative to the individuals SH risk and seizure frequency, safety of select medication (e.g., ASMs), and specific considerations for patients with FCCM.

#### Timing considerations

A neurologist or obstetrician should guide patients on the optimal timing to consider conceiving. Ideally, pregnancy should be planned when the risk of CM hemorrhage is lowest, and if the patient has seizures, they should be well-controlled. Natural history studies indicate that the risk of recurrent hemorrhage is highest within the first year following an initial event and begins to plateau around 2.5 years thereafter (16). Consequently, patients who have recently experienced SH are generally advised to wait at least 1 year before conceiving, if feasible, considering maternal age and other factors. Similarly, since frequent and/or convulsive seizures during pregnancy can negatively affect both the mother and fetus, achieving adequate seizure control prior to conception is ideal.

#### ASM management

For female patients with a history of seizures, managing ASMs involves important considerations, especially when planning for pregnancy. First, ASMs may have teratogenic potential, posing risk to the developing fetus. The fetus is most vulnerable to congenital malformations early in development, often before the pregnancy is recognized (41–43). To mitigate these risks, folate supplementation in addition to discussions on whether the ASM should be continued, held or changed to an alternative should take place. It is recommended that women of childbearing age on ASMs take at least 0.4 mg of folic acid daily, as this supplementation may help reduce the risk of neural tube defects associated with ASM use (47). Prospective registry studies indicate that valproic acid has the highest teratogenic potential, while lamotrigine and levetiracetam are associated with the lowest risk. The teratogenic effects of newer ASMs remain less well understood (57–62). Additionally, achieving optimal seizure control before pregnancy is crucial, as poorly controlled seizures can increase the risk of preterm labor and pose significant risks to the fetus. Therefore, baseline ASM levels should be obtained and maintained at effective therapeutic levels to ensure both maternal and fetal safety during pregnancy (44, 63–65).

#### Genetic counseling for FCCM

Patients with FCCM should receive genetic counseling regarding the risk of passing the mutated gene to their offspring. The genes *CCM1*, *CCM2*, and *CCM3* are inherited in an autosomal dominant manner. However, not all individuals with the gene develop CM lesions, and among those who do, symptoms may vary significantly (66, 67). Data suggests that by age 80, approximately 60.4% of patients with FCCM will experience at least one seizure (68), and up to 64% will experience at least one symptomatic cerebral or spinal hemorrhage within the first 10 years after diagnosis. It is also important to note that as many as 20% of patients may remain asymptomatic for many years (69).

#### Reproductive options and considerations

For patients with familial CCM, reproductive decisions can be complex and may require input from genetic counselors, fertility experts, and obstetricians (69). Patients may choose to proceed with natural conception, consider using donor sperm or eggs, explore *in vitro* fertilization (IVF) with or without embryo selection (70), or consider adoption. While not studied in CM patients, there is an increased risk of venous thromboembolism in patients undergoing IVF (71). Given that CM hemorrhage may be the result of intralesional thrombosis, concern has been raised about the potential for IVF medications to cause CM hemorrhage. Thus, the decision to pursue IVF should be made with careful consideration of the individual’s CM hemorrhage risk and in consultation with a healthcare provider experienced in managing CM. Genetic testing of embryos and chorionic villus sample require prior knowledge of the parental mutation. Chorionic villus sampling (CVS) for genetic testing can be performed between 10 and 12 weeks of pregnancy (72, 73).

### Risk during pregnancy

#### Concerns and recent evidence

Early case reports and series initially raised concerns that pregnancy might increase the risk of CM hemorrhage. However, more recent studies from large tertiary care centers suggest that the risk of CM hemorrhage during pregnancy may be comparable to the non-pregnant state (31–33). For example, Kalani et al. followed 64 female patients with 168 pregnancies and found that four patients experienced five hemorrhages during pregnancy, resulting in an annual hemorrhage rate of 3.4% per year pregnant. The study concluded that this rate was similar to the natural history of CM hemorrhage in non-pregnant patients, although the average age of diagnosis was not specified (31).

In a study from Toronto, Witiw and colleagues assessed 186 female patients with 349 pregnancies. They estimated the risk of CM hemorrhage by assuming the patient was at risk between the ages of 15 and 45 (childbearing years). The rate of hemorrhage during pregnancy was calculated as 1.15% per patient-year, compared to 1.01% per patient-year during the non-pregnant state. However, this methodology assumed that CM lesions were present throughout the reproductive years, despite the unclear timing of cavernoma development, potentially underestimating the risk during pregnancy, particularly given that the average age of diagnosis in the Toronto study was 42.7 years (32).

Joseph and colleagues addressed this potential confounder by assessing the risk of CM hemorrhage in female patients diagnosed with CM prior to age 46. Of the 90 patients, 136 pregnancies occurred before CM diagnosis, with four cases of CM hemorrhage during pregnancy leading to the initial diagnosis. Of 21 patients with 32 pregnancies *after* the CM diagnosis, no hemorrhages occurred during subsequent pregnancies. The rate of hemorrhage during the non-pregnant state in the same group was 10.4% per patient-year. The authors concluded that the risk of CM hemorrhage was similar in both the pregnant and non-pregnant states. However, they acknowledged the small sample size and the possibility of selection bias, as patients who experienced SH might have deferred future pregnancies, further complicating the interpretation of results (33).

#### Current recommendations and surgical considerations

Patients should be counseled that the absolute risk of complications during pregnancy is low, but close monitoring for clinical symptoms is important. Patients should be educated about their individual potential symptoms to be concerned about, which can vary depending on CM location—whether in the spine, brainstem, or cortex. Symptoms of concern, such as persistent, severe headaches or focal neurological deficits, may necessitate further investigation to rule out conditions like venous thrombosis, pre-eclampsia, or CM-related complications. MRI without contrast is considered safe during pregnancy if such concerns arise.

In the non-pregnant state, surgery is generally favored for CM patients with recurrent seizures unresponsive to medication or SH with mass effect in non-eloquent regions, as well as in cases of repeated hemorrhages and increasing disability in eloquent regions. When symptomatic or hemorrhagic CMs present with acute neurological decline, urgent surgical intervention is typically warranted (74). Most experts agree that surgical resection during pregnancy should be reserved for those with rapidly progressive symptoms and that it should be performed by a multidisciplinary team at a specialized center (29, 75–77).

#### ASM management during pregnancy

Most patients with well-controlled seizures before pregnancy will remain seizure-free during pregnancy (64). However, 20–30% of patients may experience increased seizure frequency during pregnancy. This is often due to a decline in ASM levels, which can drop significantly during pregnancy, particularly with medications like lamotrigine, levetiracetam, oxcarbazepine, topiramate, and zonisamide. This decline is attributed to changes in maternal renal and hepatic metabolism, volume of distribution, and interference by placental enzymes. Therefore, it is recommended to establish baseline ASM levels prior to pregnancy and to monitor levels throughout pregnancy, adjusting doses as necessary to maintain pre-pregnancy levels. Depending on the ASM, levels may need to be checked every 4 weeks (47), although no standardized testing frequency protocols have been published. Other factors contributing to seizure exacerbation during pregnancy may include stress, sleep deprivation, and infection. If a previously well-controlled seizure disorder worsens without an apparent cause, MRI might be considered to assess for CM hemorrhage.

### Delivery and postpartum management

#### Concerns during childbirth

Pushing during childbirth has raised concerns about the potential for intracerebral hemorrhage in patients with vascular malformations. However, there have been only rare reports of hemorrhage from a CM during delivery (58). Vaginal delivery is generally considered safe and reasonable for stable patients with CM (29). In cases where the patient has a recent neurologic issue, such as a significant focal neurologic deficit or a recent CM hemorrhage, a Cesarean section may be considered.

#### Postpartum management and breastfeeding considerations

The postpartum period, typically defined as the first 4–6 weeks after birth, requires ongoing monitoring of neurological symptoms and ASM levels, as hormonal fluctuations can persist for up to 6 months. For women whose ASM dose was increased during pregnancy, serum levels may rise shortly after delivery, necessitating dose reductions to avoid toxicity. In ASMs cleared by glucuronidation, such as lamotrigine, tapering should begin as early as 3 days postpartum, while ASMs with renal clearance may return to pre-pregnancy metabolism within 2–3 weeks postpartum (41, 44).

Breastfeeding offers numerous health benefits, but there are specific considerations for patients with CM. If an MRI is needed postpartum, it should be performed without contrast, or the patient should pump and discard breast milk for 24–48 h after the procedure. ASMs can be present in breast milk, though at lower concentrations than a fetus is exposed to during pregnancy. Studies have not demonstrated adverse effects of maternal ASM use during breastfeeding on the IQ, social or motor skills, or behavior of breastfed children (78, 79). Therefore, women on ASMs can breastfeed but should monitor their babies for signs of sedation, particularly if taking benzodiazepines or barbiturates (41).

## Mental health considerations in female patients with CM

Mental health concerns, including anxiety, depression, and fatigue, are prevalent among patients with CM, regardless of sex. These issues often arise from the uncertainty of living with a potentially hemorrhagic lesion, the impact of seizure disorders, and the burden of chronic neurological symptoms. Studies have shown that anxiety, fatigue, and impaired social and physical function are common complaints among CM patients, though these issues are not statistically different between male and female patients (80–83). Anxiety is common in patients with CM. Anxiety may relate to a new diagnosis or symptoms or can be induced by the location of the lesion. Patients may experience additional anxiety related to the risk of hemorrhage, or concerns about passing on genetic mutations in familial cases. Those with seizure disorders may also worry about the side effects of ASMs, including potential cognitive or mood changes. The influence of hormonal fluctuations on CM-related symptoms and the challenges of managing CM during pregnancy may also play a role. Counseling for patients should address these sex-and gender-specific concerns, providing strategies for managing these mental health challenges effectively. Rehabilitation programs, particularly those adapted from stroke rehabilitation protocols, may offer both physical and mental health support tailored to the needs of CM patients (84). These programs can be particularly beneficial for patients with ongoing physical dysfunction and fatigue, helping them cope with both the physical and emotional challenges associated with the condition, and ultimately improving their overall quality of life.

## Conclusion

Managing women with CM requires a comprehensive, multidisciplinary approach that addresses the unique challenges posed by hormonal influences, pregnancy, seizures, and mental health. Neurologists should take a central role in coordinating care, ensuring that all aspects of the patient’s health are considered in the management plan. This includes facilitating communication between different specialists and ensuring comprehensive management that anticipates and addresses the various challenges these patients face. Further research is needed to better understand the interactions between hormones—including estrogen, progesterone, and other sex hormones—and CM, as well as the long-term outcomes for women with these lesions in both clinical and experimental settings. Specifically, studies assessing which patient characteristics, and which hormonal therapies raise risk of SH will be impactful. In addition, further study on the influence of endogenous hormones and pregnancy could provide further insight on clinical management strategies.
